# Supplementary material for: Factors affecting bus accident severity in Thailand: A multinomial logit model
Source: PLoS One. 2022 Nov 9;17(11):e0277318. doi: 10.1371/journal.pone.0277318 (PMC9645622; doi:10.1371/journal.pone.0277318)
Supplement: Attachment — Submitted filename: Response to Reviewers2 - PlosOne_WM_18092022.docx [file pone.0277318.s002.docx]

Response to Reviewer 2 Comments

**Comments and Suggestions for Authors**

Reviewer #2:

This topic is interesting, and the whole manuscript is well organized, but it is very necessary to highlight its major contribution to the literature. I have the following issues that require particular attention in further revision:

**Point 1:**

1) It should be noted that affecting parameters on the injury severity outcomes may have temporal instability across different time periods. The authors use data from 2010 to 2019. This time period covers the great recession and other years where temporal instability of parameters is a known problem. During such a large time period that many influencing factors, such as the roadway network, population numbers, signal control conditions, speed limit standards, etc., have changed significantly, so it is not practical to disregard these changes for this study;

**Response 1**

Thank you for your kind comments. We agreed with the reviewer and added the sentence in the limitation of this study to explain the temporal instability across different time periods as following;

“Previous studies showed that affecting parameters such as the red-light running behaviors [1], individual characteristics [2], and speed limit standards [3] on the injury severity outcomes may have heterogeneity and temporal instability across different time periods that could exist in the models. As such, random parameters model such as random parameters logit model with heterogeneity [4] and Bayesian approach [1,2] could be used in further study.”

Page 13 Line 423

**Point 2:**

2. Some key indicators need to be clearly defined. For example, how do you distinguish between injury and fatality, and what are the specific criteria for morning, afternoon/evening and night? Why is it necessary to study by region (central, northern, northeast, etc.; a picture may be better to show the different region)?

**Response 2**

We agreed with the reviewer. The criteria to distinguish between injury and fatality is that the accident injury defined as people have been hospitalized at least one case, but fatality means people have died by the bus accident at least one death. The specific criteria for morning, afternoon/evening and night are that morning is defined as 6.00-11.59 a.m., afternoon and evening (12.00 p.m.-5.59 p.m.), and night (6.00 p.m.-5.59 a.m.). Those categories depend on traffic volume. Moreover, it is necessary to study by region (central, northern, northeast, etc.) because the bus accident varies across the region. We have further reviewed and revised the explanation of the severity outcome, time periods, and region.

Therefore, we added the sentence as follow:

“A ‘non-injury’ means an accident which leads to property damage only without any injury cases. An ‘injury’ means an accident which leads to the injury and needs hospitalization of a victim on the bus at least one case. A 'fatal accident' means an accident which leads to the death of a victim at least one death.”

Page 6 Line 167

We also added the Thai map to show the different regions as follow:

“Therefore, the accidents in this study were divided into six regions: Central, Northern, Northeast, Eastern, Western, and Southern as shown in Fig 1.”

Fig 1. Map of Thailand illustrating 6 regions.

Page 5 Line 148

**Point 3:**

3) Analysis of marginal effects is lacking and is recommended to be added;

**Response 3:**

We agreed with the reviewer and analysed the marginal effects as following;

“Marginal effects were computed and used to assess the effect of each variable on the bus accident severity outcome probabilities.”

Page 7 Line 214

“The results of the marginal effects for bus accidents in Thailand are given in Table 4. Drunk driving greatly increases the probability of fatal injury by 42.4% in bus accidents compared to accidents occurring in non-drunk driving. While, wrong-way driving direction increases the probability of injury by 18.2%. In terms of crash characteristics, the crashes occurring in Northern Thailand increases the probability of fatal injury by 11.1% in bus accidents compared to accidents occurring in Central region. Other crash characteristic variables show that the crashes occurring in Northeast Thailand increases the probability of injury by 10.5% compared to accidents occurring in Central region. Environmental characteristics were also found to be significant. Curve roads was found to significantly increase the probability of accident fatality by 8.7% compared to straight roads. While, sloped roads were found to significantly increase the probability of injury by 17.2% compared to flat roads.

Table 4

The results of the marginal effects for bus accidents in Thailand

| Variables | Non-injury | Injury | Fatality |
| --- | --- | --- | --- |
| Bus driver factors | | | |
| Not obeying traffic rules | -11.4% | -8.4% | 19.7% |
| Abrupt driving | -11.5% | -1.5% | 13.1% |
| Drunk driving | -11.5% | -30.9% | 42.4% |
| Fallen asleep | -22.5% | 10.9% | 11.1% |
| Right-way driving direction | -10.0% | 10.7% | -0.7% |
| Wrong-way driving direction | -17.2% | 18.2% | -1.0% |
| Crash characteristics | | | |
| Afternoon/evening | 6.3% | -2.5% | -0.4% |
| Northern region | -19.4% | 8.4% | 11.1% |
| Northeast region | -18.5% | 10.5% | 8.1% |
| Eastern region | -9.9% | 2.8% | 7.1% |
| Western region | -17.8% | 7.2% | 10.6% |
| Southern region | -18.0% | 8.8% | 9.2% |
| Environmental characteristics | | | |
| Highways without frontage roads | -15.9% | 7.5% | 8.3% |
| Curve roads | -6.6% | -2.1% | 8.7% |
| Sloped roads | -22.6% | 17.2% | 5.5% |
| Median openings | -7.9% | 4.1% | 3.9% |
| Road junctions | -16.0% | 14.3% | 1.7% |

Page 11 Line 307

**Point 4:**

4) What type of road does highway in Table 2 specifically refer to? This in turn suggests to us that the research object in this study, bus, refers only to that operating on urban roads? All these details need to be spelled out;

**Response 4:**

We agreed with the reviewer and explained the definition of the highway in Thailand which refers to that operating on urban and rural roads. Highways are main roads allocated for the transportation of people or goods that operating on urban and rural roads. It connects different regions, provinces, and districts.

Therefore, we added the sentences to define the highway as following;

“Highways are main roads allocated for the transportation of people or goods that operating on urban and rural roads. It connects different regions, provinces, and districts [5]. ”

Page 3 Line 110

**Point 5:**

5) In the discussion section, it is suggested that the authors further suggest relevant preventive policy measures for bus accidents;

**Response 5:**

We agreed with the reviewer and added the relevant preventive policy measures for bus accidents in the discussion section as following;

“To prevent the bus driver risk factors, previous studies showed that awareness campaigns in reducing the accidents on highways should be implemented [6] and the driver should be encouraged to register the driver licensing [7].”

Page 13 Line 383

“Previous studies showed that rescheduling of rush hour driving could reduce the incidence of crashes [8] and avoiding unfamiliar place could be reduced the risk of accident [9].”

Page 13 Line 397

“To prevent bus accident severity, previous studies suggested that policy recommendations for improving road conditions should be proposed [10] and road authorities should concern the safety on the high risk roads [11-13].”

Page 13 Line 415

**Point 6:**

6) It is recommended that one form be retained for the number or percentage of accidents in Table 2; both p-values and asterisks are given in Table 3, and the information on both is repeated, so it is recommended that one form be retained.

**Response 6:**

We agreed with the reviewer, however in table2, we presented both frequencies and percentage because it would be benefit for reader, in case, they need to recalculate the the frequencies to percentage in both row and column. While we deleted the asterisks in Table 3 to avoid the repeat of p-value.

Page 10 Line 290

Reference:

1.	Guo, Y.; Li, Z.; Wu, Y.; Xu, C. Exploring unobserved heterogeneity in bicyclists’ red-light running behaviors at different crossing facilities. *Accident Analysis & Prevention* **2018**, *115*, 118-127, doi:<https://doi.org/10.1016/j.aap.2018.03.006>.

2.	Guo, Y.; Li, Z.; Wu, Y.; Xu, C. Evaluating factors affecting electric bike users’ registration of license plate in China using Bayesian approach. *Transportation Research Part F: Traffic Psychology and Behaviour* **2018**, *59*, 212-221, doi:<https://doi.org/10.1016/j.trf.2018.09.008>.

3.	Alnawmasi, N.; Mannering, F. A statistical assessment of temporal instability in the factors determining motorcyclist injury severities. *Analytic Methods in Accident Research* **2019**, *22*, 100090, doi:<https://doi.org/10.1016/j.amar.2019.100090>.

4.	Xie, P.; Qin, D.; Zhu, T. Impact of rule-violating behaviors on the risk of bus drivers being at-fault in crashes. *Traffic Inj Prev* **2022**, *23*, 364-368, doi:10.1080/15389588.2022.2079639.

5.	Champahom, T.; Jomnonkwao, S.; Banyong, C.; Nambulee, W.; Karoonsoontawong, A.; Ratanavaraha, V. Analysis of Crash Frequency and Crash Severity in Thailand: Hierarchical Structure Models Approach. *Sustainability* **2021**, *13*, doi:10.3390/su131810086.

6.	Gopalakrishnan, S. A public health perspective of road traffic accidents. *J Family Med Prim Care* **2012**, *1*, 144-150, doi:10.4103/2249-4863.104987.

7.	Hartling, L.; Wiebe, N.; Russell, K.; Petruk, J.; Spinola, C.; Klassen, T.P. Graduated driver licensing for reducing motor vehicle crashes among young drivers. *Cochrane Database Syst Rev* **2004**, 10.1002/14651858.CD003300.pub2, Cd003300, doi:10.1002/14651858.CD003300.pub2.

8.	Wang, S.Y.; Wu, K.F. Reducing intercity bus crashes through driver rescheduling. *Accid Anal Prev* **2019**, *122*, 25-35, doi:10.1016/j.aap.2018.09.019.

9.	Stewart, B.T.; Yankson, I.K.; Afukaar, F.; Medina, M.C.; Cuong, P.V.; Mock, C. Road Traffic and Other Unintentional Injuries Among Travelers to Developing Countries. *Med Clin North Am* **2016**, *100*, 331-343, doi:10.1016/j.mcna.2015.07.011.

10.	Nguyen, T.C.; Nguyen, M.H.; Armoogum, J.; Ha, T.T. Bus Crash Severity in Hanoi, Vietnam. *Safety* **2021**, *7*, doi:10.3390/safety7030065.

11.	Abdollahzadeh Nasiri, A.S.; Rahmani, O.; Abdi Kordani, A.; Karballaeezadeh, N.; Mosavi, A. Evaluation of Safety in Horizontal Curves of Roads Using a Multi-Body Dynamic Simulation Process. *Int J Environ Res Public Health* **2020**, *17*, doi:10.3390/ijerph17165975.

12.	Jones, A.P.; Haynes, R.; Harvey, I.M.; Jewell, T. Road traffic crashes and the protective effect of road curvature over small areas. *Health & Place* **2012**, *18*, 315-320, doi:<https://doi.org/10.1016/j.healthplace.2011.10.008>.

13.	Polus, A.; Pollatschek, M.A.; Farah, H. Impact of infrastructure characteristics on road crashes on two-lane highways. *Traffic Inj Prev* **2005**, *6*, 240-247, doi:10.1080/15389580590969210.
