# Supplementary material for: Factors affecting bus accident severity in Thailand: A multinomial logit model
Source: PLoS One. 2022 Nov 9;17(11):e0277318. doi: 10.1371/journal.pone.0277318 (PMC9645622; doi:10.1371/journal.pone.0277318)
Supplement: Attachment — Submitted filename: Response to Reviewers2 - PlosOne_WM_20102022.docx [file pone.0277318.s003.docx]

Response to Reviewer 2 Comments

**Comments and Suggestions for Authors**

Reviewer #2:

First of all, thank you for your response to the questions I asked previously. I think the current version has been upgraded more fully and is closer to the level of publication, and in order to promote readability, I have the following small suggestions:

**Point 1:**

1) The quantities and percentages in Table 2 are repetitive expressions of the same information and it is recommended that one presentation be retained. Also, please be careful with the use of percentage signs; it is very uncomfortable not to have the whole table look like a percentage sign;

**Response 1**

Thank you for your kind comments. We agreed with the reviewer. We deleted the quantities (number of accidents) and percentage signs in Table 2 as following;

**Table 2**

Descriptive statistics (%) of risk factors from 2010 to 2019 in Thailand.

| Variables | Non-injury | Injury | Fatality | Total |
| --- | --- | --- | --- | --- |
| Bus driver factors |  |  |  |  |
| Excessive speed |  |  |  |  |
| No^a^ | 32.1 | 40.6 | 27.3 | 31.5 |
| Yes | 47.0 | 38.1 | 14.9 | 68.5 |
| Not obeying traffic rules |  |  |  |  |
| No^a^ | 42.9 | 39.2 | 17.9 | 94.8 |
| Yes | 30.9 | 32.9 | 36.2 | 5.2 |
| Abrupt driving |  |  |  |  |
| No^a^ | 43.4 | 38.9 | 17.7 | 88.3 |
| Yes | 33.5 | 39.1 | 27.4 | 11.7 |
| Drunk driving |  |  |  |  |
| No^a^ | 42.3 | 40.0 | 18.7 | 99.7 |
| Yes | 30.0 | 10.0 | 60.0 | 0.34 |
| Fallen asleep |  |  |  |  |
| No^a^ | 43.6 | 38.0 | 18.4 | 94.4 |
| Yes | 20.9 | 53.4 | 25.7 | 5.6 |
| Driving direction |  |  |  |  |
| Unknown ^a^ | 55.9 | 27.5 | 16.6 | 13.9 |
| Right-way | 41.7 | 38.8 | 19.5 | 59.1 |
| Wrong-way | 36.5 | 44.9 | 18.6 | 27.0 |
|  |  |  |  |  |
| Crash characteristics |  |  |  |  |
| Month |  |  |  |  |
| January^a^ | 40.5 | 40.9 | 18.6 | 11.3 |
| February | 43.8 | 36.4 | 19.8 | 7.4 |
| March | 40.4 | 36.5 | 23.1 | 8.9 |
| April | 40.9 | 40.5 | 18.6 | 13.8 |
| May | 48.1 | 35.7 | 16.2 | 7.4 |
| June | 47.8 | 38.5 | 13.7 | 6.3 |
| July | 41.1 | 40.1 | 18.8 | 6.9 |
| August | 43.7 | 38.0 | 18.3 | 7.7 |
| September | 52.7 | 35.5 | 11.8 | 5.8 |
| October | 39.1 | 38.1 | 22.8 | 6.8 |
| November | 43.6 | 40.4 | 16.0 | 7.5 |
| December | 33.9 | 42.4 | 23.7 | 10.1 |
| Time of day |  |  |  |  |
| Morning^a^ | 40.2 | 41.0 | 18.8 | 50.4 |
| Afternoon/evening | 47.2 | 37.7 | 15.1 | 26.1 |
| Night | 41.5 | 35.6 | 22.9 | 23.5 |
| Region |  |  |  |  |
| Central^a^ | 57.2 | 31.6 | 11.2 | 44.5 |
| Northern | 25.4 | 44.0 | 30.6 | 8.0 |
| Northeast | 30.3 | 46.5 | 23.3 | 22.5 |
| Eastern | 40.1 | 39.2 | 20.7 | 7.8 |
| Western | 26.1 | 46.6 | 27.3 | 5.5 |
| Southern | 29.1 | 45.0 | 25.9 | 11.7 |
| Environmental characteristics |  |  |  |  |
| Highways without a frontage road |  |  |  |  |
| No^a^ | 60.2 | 30.9 | 8.9 | 25.4 |
| Yes | 36.2 | 41.6 | 22.2 | 74.6 |
| Road horizontal alignment |  |  |  |  |
| Straight^a^ | 44.9 | 38.5 | 16.6 | 85.5 |
| Curved | 27.4 | 41.0 | 31.6 | 13.4 |
| Sharply curved | 18.2 | 45.4 | 36.4 | 1.1 |
| Road vertical alignment |  |  |  |  |
| Flat^a^ | 44.1 | 38.3 | 17.6 | 92.6 |
| Upwardly curved | 25.0 | 33.9 | 41.1 | 1.9 |
| Downwardly curved | 27.8 | 33.3 | 38.9 | 1.2 |
| Sloped | 15.2 | 56.0 | 28.8 | 4.3 |
| Intersection type |  |  |  |  |
| No Intersection^a^ | 43.3 | 38.9 | 17.8 | 88.5 |
| Four-way | 33.8 | 40.0 | 27.2 | 4.7 |
| T-shaped | 34.9 | 38.8 | 26.3 | 4.4 |
| Y-shaped | 26.9 | 38.5 | 34.6 | 0.9 |
| Others | 37.8 | 37.8 | 24.4 | 1.5 |
| Median opening |  |  |  |  |
| No^a^ | 43.1 | 38.5 | 18.4 | 92.6 |
| Yes | 31.9 | 43.5 | 24.5 | 7.4 |
| Road junctions |  |  |  |  |
| No^a^ | 42.7 | 38.6 | 18.7 | 98.1 |
| Yes | 22.2 | 51.9 | 25.9 | 1.9 |

This table displays percentage. The total column contains percentages calculated across rows within one variable. The injury severity columns contain percentages calculated across columns within one row.

^a^ Indicates referent category.

Page 7 Line 238

**Point 2:**

2. 0.1567 in the first row of Table 3 should be taken as 0.157;

**Response 2**

Thank you for your suggestions. We revised the value from 0.1567 in the first row of Table 3 to 0.157.

Page 10 Line 1213

**Point 3:**

3) Please deal with the percentage signs in Table 4, the whole table is now about percentages and the same expression looks very uncomfortable;

**Response 3:**

We agreed with the reviewer and deleted the percentage signs in Table 4 as following;

**Table 4**

The results of the marginal effects for bus accidents in Thailand (%)

| Variables | Non-injury | Injury | Fatality |
| --- | --- | --- | --- |
| Bus driver factors | | | |
| Not obeying traffic rules | -11.4 | -8.4 | 19.7 |
| Abrupt driving | -11.5 | -1.5 | 13.1 |
| Drunk driving | -11.5 | -30.9 | 42.4 |
| Fallen asleep | -22.5 | 10.9 | 11.1 |
| Right-way driving direction | -10.0 | 10.7 | -0.7 |
| Wrong-way driving direction | -17.2 | 18.2 | -1.0 |
| Crash characteristics | | | |
| Afternoon/evening | 6.3 | -2.5 | -0.4 |
| Northern region | -19.4 | 8.4 | 11.1 |
| Northeast region | -18.5 | 10.5 | 8.1 |
| Eastern region | -9.9 | 2.8 | 7.1 |
| Western region | -17.8 | 7.2 | 10.6 |
| Southern region | -18.0 | 8.8 | 9.2 |
| Environmental characteristics | | | |
| Highways without frontage roads | -15.9 | 7.5 | 8.3 |
| Curve roads | -6.6 | -2.1 | 8.7 |
| Sloped roads | -22.6 | 17.2 | 5.5 |
| Median openings | -7.9 | 4.1 | 3.9 |
| Road junctions | -16.0 | 14.3 | 1.7 |

Page 11 Line 307

**Point 4:**

4) Please provide information on whether the data is publicly available;

**Response 4:**

Thank you for your recommendation. We provided the information on whether the data is publicly available as following;

“Data Availability

All relevant data are within the paper and its Supporting information files.”

Page 11 Line 1231

**Point 5:**

5) Further changes to the format of the references are required.

**Response 5:**

Thank you for your recommendation. We revised the format of the references using PLOS style in the main text as following;

1.	Kaplan S, Prato CG. Risk factors associated with bus accident severity in the United States: a generalized ordered logit model. J Safety Res. 2012;43(3):171-80. Epub 2012/09/15. doi: 10.1016/j.jsr.2012.05.003. PubMed PMID: 22974682.

2.	Morency P, Strauss J, Pépin F, Tessier F, Grondines J. Traveling by Bus Instead of Car on Urban Major Roads: Safety Benefits for Vehicle Occupants, Pedestrians, and Cyclists. J Urban Health. 2018;95(2):196-207. doi: 10.1007/s11524-017-0222-6. PubMed PMID: 29500736.

3.	Pearce T, Maunder DAC, Mbara TC, Babu DM, Rwebangira T. Bus Accidents in India, Nepal, Tanzania, and Zimbabwe. Transportation Research Record. 2000;1726(1):16-23. doi: 10.3141/1726-03.

4.	Department of Land Transport. Bus accident data https://web.dlt.go.th/statistics/index.php2021 [cited October 14, 2021]. Available from: https://web.dlt.go.th/statistics/index.php.

5.	WHO. Global Status Report on Road Safety 2018 https://www.who.int/thailand/news/detail/19-12-2018-launch-of-the-global-status-report-on-road-safety-2018-in-thailand2018 [cited October 14, 2021]. Available from: https://www.who.int/thailand/news/detail/19-12-2018-launch-of-the-global-status-report-on-road-safety-2018-in-thailand.

6.	Feng S, Li Z, Ci Y, Zhang G. Risk factors affecting fatal bus accident severity: Their impact on different types of bus drivers. Accid Anal Prev. 2016;86:29-39. Epub 2015/10/30. doi: 10.1016/j.aap.2015.09.025. PubMed PMID: 26513334.

7.	Barua U, Tay R. Severity of urban transit bus crashes in Bangladesh. Journal of Advanced Transportation. 2010;44(1):34-41. doi: https://doi.org/10.1002/atr.104.

8.	Anund A, Dukic T, Thornthwaite S, Falkmer T. Is European school transport safe?—The need for a “door-to-door” perspective. European Transport Research Review. 2011;3(2):75-83. doi: 10.1007/s12544-011-0052-7.

9.	Tetali S, Edwards P, Murthy GVS, Roberts I. Road traffic injuries to children during the school commute in Hyderabad, India: cross-sectional survey. Injury Prevention. 2016;22(3):171. doi: 10.1136/injuryprev-2015-041854.

10.	Chimba D, Sando T, Kwigizile V. Effect of bus size and operation to crash occurrences. Accident Analysis & Prevention. 2010;42(6):2063-7. doi: https://doi.org/10.1016/j.aap.2010.06.018.

11.	Razmpa E, Sadegh Niat K, Saedi B. Urban bus drivers' sleep problems and crash accidents. Indian J Otolaryngol Head Neck Surg. 2011;63(3):269-73. Epub 2012/07/04. doi: 10.1007/s12070-011-0235-5. PubMed PMID: 22754808; PubMed Central PMCID: PMCPMC3138945.

12.	Blower D, Green P. Type of Motor Carrier and Driver History in Fatal Bus Crashes. Transportation Research Record: Journal of the Transportation Research Board. 2010;2194. doi: 10.3141/2194-05.

13.	af Wåhlberg AE. Characteristics of low speed accidents with buses in public transport: part II. Accid Anal Prev. 2004;36(1):63-71. Epub 2003/10/24. doi: 10.1016/s0001-4575(02)00128-8. PubMed PMID: 14572828.

14.	af Wåhlberg AE. Speed choice versus celeration behavior as traffic accident predictor. J Safety Res. 2006;37(1):43-51. Epub 2006/02/28. doi: 10.1016/j.jsr.2005.10.017. PubMed PMID: 16499929.

15.	Af Wåhlberg AE. The relation of non-culpable traffic incidents to bus drivers' celeration behavior. J Safety Res. 2008;39(1):41-6. Epub 2008/03/08. doi: 10.1016/j.jsr.2007.10.009. PubMed PMID: 18325415.

16.	Damsere-Derry J, Adanu EK, Ojo TK, Sam EF. Injury-severity analysis of intercity bus crashes in Ghana: A random parameters multinomial logit with heterogeneity in means and variances approach. Accid Anal Prev. 2021;160:106323. Epub 2021/08/12. doi: 10.1016/j.aap.2021.106323. PubMed PMID: 34380083.

17.	Jayatilleke AU, Nakahara S, Dharmaratne SD, Jayatilleke AC, Poudel KC, Jimba M. Working conditions of bus drivers in the private sector and bus crashes in Kandy district, Sri Lanka: a case-control study. Inj Prev. 2009;15(2):80-6. Epub 2009/04/07. doi: 10.1136/ip.2008.018937. PubMed PMID: 19346419.

18.	Chaiard J, Deeluea J, Suksatit B, Songkham W. Factors associated with sleep quality of Thai intercity bus drivers. Industrial Health. 2019;57(5):596-603. doi: 10.2486/indhealth.2018-0168.

19.	Prato CG, Kaplan S. Bus accident severity and passenger injury: evidence from Denmark. European Transport Research Review. 2014;6(1):17-30. doi: 10.1007/s12544-013-0107-z.

20.	Ponnaluri RV. The odds of wrong-way crashes and resulting fatalities: A comprehensive analysis. Accident Analysis & Prevention. 2016;88:105-16. doi: https://doi.org/10.1016/j.aap.2015.12.012.

21.	Riyapan S, Thitichai P, Chaisirin W, Nakornchai T, Chakorn T. Outcomes of Emergency Medical Service Usage in Severe Road Traffic Injury during Thai Holidays. West J Emerg Med. 2018;19(2):266-75. Epub 2018/02/20. doi: 10.5811/westjem.2017.11.35169. PubMed PMID: 29560053.

22.	Klinjun N, Lim A, Bundhamcharoen K. Epidemiologic patterns of transport accident mortality in Thailand. Southeast Asian J Trop Med Public Health. 2016;47(2):318-27. Epub 2016/06/02. PubMed PMID: 27244970.

23.	Abrari Vajari M, Aghabayk K, Sadeghian M, Shiwakoti N. A multinomial logit model of motorcycle crash severity at Australian intersections. Journal of Safety Research. 2020;73:17-24. doi: https://doi.org/10.1016/j.jsr.2020.02.008.

24.	Khorshidi A, Ainy E, Hashemi Nazari SS, Soori H. Temporal Patterns of Road Traffic Injuries in Iran. Arch Trauma Res. 2016;5(2):e27894. Epub 2016/10/06. doi: 10.5812/atr.27894. PubMed PMID: 27703958; PubMed Central PMCID: PMCPMC5037289.

25.	Shahla F, Shalaby AS, Persaud BN, Hadayeghi A. Analysis of Transit Safety at Signalized Intersections in Toronto, Ontario, Canada. Transportation Research Record. 2009;2102(1):108-14. doi: 10.3141/2102-14.

26.	Nguyen TC, Nguyen MH, Armoogum J, Ha TT. Bus Crash Severity in Hanoi, Vietnam. Safety. 2021;7(3). doi: 10.3390/safety7030065.

27.	Ahmed M, Khanom K, Shampa RM, Bari MH. Road traffic accident among motor vehicle drivers in selected high ways. Mymensingh Med J. 2004;13(2):165-8. Epub 2004/07/31. PubMed PMID: 15284694.

28.	Yang J, Peek-Asa C, Cheng G, Heiden E, Falb S, Ramirez M. Incidence and characteristics of school bus crashes and injuries. Accident Analysis & Prevention. 2009;41(2):336-41. doi: https://doi.org/10.1016/j.aap.2008.12.012.

29.	Abellán J, López G, de Oña J. Analysis of traffic accident severity using Decision Rules via Decision Trees. Expert Systems with Applications. 2013;40(15):6047-54. doi: https://doi.org/10.1016/j.eswa.2013.05.027.

30.	de Lapparent M. Empirical Bayesian analysis of accident severity for motorcyclists in large French urban areas. Accident Analysis & Prevention. 2006;38(2):260-8. doi: https://doi.org/10.1016/j.aap.2005.09.001.

31.	Department of Highways. Bus accident data https://www.doh.go.th/visual/accident2021 [cited October 14, 2021]. Available from: https://www.doh.go.th/visual/accident.

32.	Champahom T, Jomnonkwao S, Banyong C, Nambulee W, Karoonsoontawong A, Ratanavaraha V. Analysis of Crash Frequency and Crash Severity in Thailand: Hierarchical Structure Models Approach. Sustainability. 2021;13(18). doi: 10.3390/su131810086.

33.	Zimmerman K, Mzige AA, Kibatala PL, Museru LM, Guerrero A. Road traffic injury incidence and crash characteristics in Dar es Salaam: a population based study. Accid Anal Prev. 2012;45:204-10. Epub 2012/01/25. doi: 10.1016/j.aap.2011.06.018. PubMed PMID: 22269502.

34.	Musa MF, Hassan SA, Mashros N. The impact of roadway conditions towards accident severity on federal roads in Malaysia. PLoS One. 2020;15(7):e0235564-e. doi: 10.1371/journal.pone.0235564. PubMed PMID: 32628689.

35.	Chen Y, Wang K, King M, He J, Ding J, Shi Q, et al. Differences in Factors Affecting Various Crash Types with High Numbers of Fatalities and Injuries in China. PLoS One. 2016;11(7):e0158559. doi: 10.1371/journal.pone.0158559.

36.	Wang Z, Huang B, Lu J, Zhao J, Zhang Y. Exploring the Impact of Access Designs on Crash Injury Severity on Multilane Highways. Traffic Injury Prevention. 2014;15(1):102-9. doi: 10.1080/15389588.2013.789871.

37.	Vorko-Jović A, Kern J, Biloglav Z. Risk factors in urban road traffic accidents. J Safety Res. 2006;37(1):93-8. Epub 2006/03/07. doi: 10.1016/j.jsr.2005.08.009. PubMed PMID: 16516927.

38.	Islam S, Mannering F. Driver aging and its effect on male and female single-vehicle accident injuries: Some additional evidence. Journal of Safety Research. 2006;37(3):267-76. doi: https://doi.org/10.1016/j.jsr.2006.04.003.

39.	Savolainen P, Mannering F. Probabilistic models of motorcyclists’ injury severities in single- and multi-vehicle crashes. Accident Analysis & Prevention. 2007;39(5):955-63. doi: https://doi.org/10.1016/j.aap.2006.12.016.

40.	Geedipally SR, Turner PA, Patil S. Analysis of Motorcycle Crashes in Texas with Multinomial Logit Model. Transportation Research Record. 2011;2265(1):62-9. doi: 10.3141/2265-07.

41.	Çelik AK, Oktay E. A multinomial logit analysis of risk factors influencing road traffic injury severities in the Erzurum and Kars Provinces of Turkey. Accident Analysis & Prevention. 2014;72:66-77. doi: https://doi.org/10.1016/j.aap.2014.06.010.

42.	Salum JH, Kitali AE, Bwire H, Sando T, Alluri P. Severity of motorcycle crashes in Dar es Salaam, Tanzania. Traffic Injury Prevention. 2019;20(2):189-95. doi: 10.1080/15389588.2018.1544706.

43.	Withers SD. Data Analysis, Categorical. In: Kobayashi A, editor. International Encyclopedia of Human Geography (Second Edition). Oxford: Elsevier; 2009. p. 159-65.

44.	Hashimoto EM, Ortega EMM, Cordeiro GM, Suzuki AK, Kattan MW. The multinomial logistic regression model for predicting the discharge status after liver transplantation: estimation and diagnostics analysis. J Appl Stat. 2020;47(12):2159-77. Epub 2019/12/24. doi: 10.1080/02664763.2019.1706725. PubMed PMID: 35706842; PubMed Central PMCID: PMCPMC9041638.

45.	Rifaat SM, Tay R, de Barros A. Effect of street pattern on the severity of crashes involving vulnerable road users. Accident Analysis & Prevention. 2011;43(1):276-83. doi: https://doi.org/10.1016/j.aap.2010.08.024.

46.	Savolainen PT, Mannering FL, Lord D, Quddus MA. The statistical analysis of highway crash-injury severities: A review and assessment of methodological alternatives. Accident Analysis & Prevention. 2011;43(5):1666-76. doi: https://doi.org/10.1016/j.aap.2011.03.025.

47.	Hausman J, McFadden D. Specification Tests for the Multinomial Logit Model. Econometrica. 1984;52(5):1219-40. doi: 10.2307/1910997.

48.	Yagoub U, Saiyed NS, Rahim B-EEA, Musawa N, Al Zahrani AM. Road Traffic Injuries and Related Safety Measures: A Multicentre Analysis at Military Hospitals in Tabuk, Saudi Arabia. Emerg Med Int. 2021;2021:6617381-. doi: 10.1155/2021/6617381. PubMed PMID: 33708446.

49.	Sam EF, Daniels S, Brijs K, Brijs T, Wets G. Modelling public bus/minibus transport accident severity in Ghana. Accid Anal Prev. 2018;119:114-21. Epub 2018/07/18. doi: 10.1016/j.aap.2018.07.008. PubMed PMID: 30016751.

50.	CTN NEWS. Thailand’s Transport Ministry to Ban Bus Drivers from Alcohol Consumption 2017 [27 April 2022]. Available from: https://www.chiangraitimes.com/news-asia-thailand/thailands-transport-ministry-to-ban-bus-drivers-from-alcohol-consumption/.

51.	Hussain Q, Feng H, Grzebieta R, Brijs T, Olivier J. The relationship between impact speed and the probability of pedestrian fatality during a vehicle-pedestrian crash: A systematic review and meta-analysis. Accid Anal Prev. 2019;129:241-9. Epub 2019/06/09. doi: 10.1016/j.aap.2019.05.033. PubMed PMID: 31176144.

52.	Sabbagh-Ehrlich S, Friedman L, Richter ED. Working conditions and fatigue in professional truck drivers at Israeli ports. Inj Prev. 2005;11(2):110-4. Epub 2005/04/05. doi: 10.1136/ip.2004.007682. PubMed PMID: 15805441; PubMed Central PMCID: PMCPMC1730197.

53.	Anund A, Ihlström J, Fors C, Kecklund G, Filtness A. Factors associated with self-reported driver sleepiness and incidents in city bus drivers. Industrial health. 2016;54(4):337-46. Epub 2016/04/19. doi: 10.2486/indhealth.2015-0217. PubMed PMID: 27098307.

54.	Rey de Castro J, Gallo J, Loureiro H. [Tiredness and sleepiness in bus drivers and road accidents in Peru: a quantitative study]. Rev Panam Salud Publica. 2004;16(1):11-8. Epub 2004/08/31. doi: 10.1590/s1020-49892004000700002. PubMed PMID: 15333261.

55.	Kemel E. Wrong-way driving crashes on French divided roads. Accid Anal Prev. 2015;75:69-76. Epub 2014/12/03. doi: 10.1016/j.aap.2014.11.002. PubMed PMID: 25460093.

56.	Gopalakrishnan S. A public health perspective of road traffic accidents. J Family Med Prim Care. 2012;1(2):144-50. Epub 2012/07/01. doi: 10.4103/2249-4863.104987. PubMed PMID: 24479025; PubMed Central PMCID: PMCPMC3893966.

57.	Hartling L, Wiebe N, Russell K, Petruk J, Spinola C, Klassen TP. Graduated driver licensing for reducing motor vehicle crashes among young drivers. Cochrane Database Syst Rev. 2004;(2):Cd003300. Epub 2004/04/24. doi: 10.1002/14651858.CD003300.pub2. PubMed PMID: 15106200.

58.	Abdul Manan MM, Várhelyi A, Çelik AK, Hashim HH. Road characteristics and environment factors associated with motorcycle fatal crashes in Malaysia. IATSS Research. 2018;42(4):207-20. doi: https://doi.org/10.1016/j.iatssr.2017.11.001.

59.	Dechkamfoo C, Sitthikankun S, Kridakorn Na Ayutthaya T, Manokeaw S, Timprae W, Tepweerakun S, et al. Impact of Rainfall-Induced Landslide Susceptibility Risk on Mountain Roadside in Northern Thailand. Infrastructures. 2022;7(2). doi: 10.3390/infrastructures7020017.

60.	Wang SY, Wu KF. Reducing intercity bus crashes through driver rescheduling. Accid Anal Prev. 2019;122:25-35. Epub 2018/10/10. doi: 10.1016/j.aap.2018.09.019. PubMed PMID: 30300796.

61.	Stewart BT, Yankson IK, Afukaar F, Medina MC, Cuong PV, Mock C. Road Traffic and Other Unintentional Injuries Among Travelers to Developing Countries. Med Clin North Am. 2016;100(2):331-43. Epub 2016/02/24. doi: 10.1016/j.mcna.2015.07.011. PubMed PMID: 26900117; PubMed Central PMCID: PMCPMC4764791.

62.	Milton JC, Shankar VN, Mannering FL. Highway accident severities and the mixed logit model: an exploratory empirical analysis. Accid Anal Prev. 2008;40(1):260-6. Epub 2008/01/25. doi: 10.1016/j.aap.2007.06.006. PubMed PMID: 18215557.

63.	Buddhavarapu P, Banerjee A, Prozzi JA. Influence of pavement condition on horizontal curve safety. Accid Anal Prev. 2013;52:9-18. Epub 2013/01/10. doi: 10.1016/j.aap.2012.12.010. PubMed PMID: 23298704.

64.	Chang F, Li M, Xu P, Zhou H, Haque MM, Huang H. Injury Severity of Motorcycle Riders Involved in Traffic Crashes in Hunan, China: A Mixed Ordered Logit Approach. Int J Environ Res Public Health. 2016;13(7):714. doi: 10.3390/ijerph13070714. PubMed PMID: 27428987.

65.	Zhao XG, He XD, Wu JS, Zhao GF, Ma YF, Zhang M, et al. Risk factors for urban road traffic injuries in Hangzhou, China. Arch Orthop Trauma Surg. 2009;129(4):507-13. Epub 2009/02/18. doi: 10.1007/s00402-009-0827-7. PubMed PMID: 19221774.

66.	Abdollahzadeh Nasiri AS, Rahmani O, Abdi Kordani A, Karballaeezadeh N, Mosavi A. Evaluation of Safety in Horizontal Curves of Roads Using a Multi-Body Dynamic Simulation Process. Int J Environ Res Public Health. 2020;17(16). Epub 2020/08/23. doi: 10.3390/ijerph17165975. PubMed PMID: 32824601; PubMed Central PMCID: PMCPMC7459981.

67.	Jones AP, Haynes R, Harvey IM, Jewell T. Road traffic crashes and the protective effect of road curvature over small areas. Health & Place. 2012;18(2):315-20. doi: https://doi.org/10.1016/j.healthplace.2011.10.008.

68.	Polus A, Pollatschek MA, Farah H. Impact of infrastructure characteristics on road crashes on two-lane highways. Traffic Inj Prev. 2005;6(3):240-7. Epub 2005/08/10. doi: 10.1080/15389580590969210. PubMed PMID: 16087465.

69.	Guo Y, Li Z, Wu Y, Xu C. Exploring unobserved heterogeneity in bicyclists’ red-light running behaviors at different crossing facilities. Accident Analysis & Prevention. 2018;115:118-27. doi: https://doi.org/10.1016/j.aap.2018.03.006.

70.	Guo Y, Li Z, Wu Y, Xu C. Evaluating factors affecting electric bike users’ registration of license plate in China using Bayesian approach. Transportation Research Part F: Traffic Psychology and Behaviour. 2018;59:212-21. doi: https://doi.org/10.1016/j.trf.2018.09.008.

71.	Alnawmasi N, Mannering F. A statistical assessment of temporal instability in the factors determining motorcyclist injury severities. Analytic Methods in Accident Research. 2019;22:100090. doi: https://doi.org/10.1016/j.amar.2019.100090.

72.	Xie P, Qin D, Zhu T. Impact of rule-violating behaviors on the risk of bus drivers being at-fault in crashes. Traffic Inj Prev. 2022;23(6):364-8. Epub 2022/06/08. doi: 10.1080/15389588.2022.2079639. PubMed PMID: 35670548.

Page 16 Line 1545
